# Supplementary material for: Baseline Characterization of the Gut Microbiota of Field and Colony Populations of Phlebotomus tobbi and Preliminary Assessment of the Anti-Leishmanial Activity of Cultivable Bacteria
Source: Pathogens. 2026 Jun 23;15(7):658. doi: 10.3390/pathogens15070658 (PMC13416292; doi:10.3390/pathogens15070658)
Supplement: Supplementary file 1 [file pathogens-15-00658-s001.zip › pathogens-4357168-File S1/Taxa2ASV_Decomposer/Family/f__Staphylococcaceae/heatmap_without_clustering.pdf]

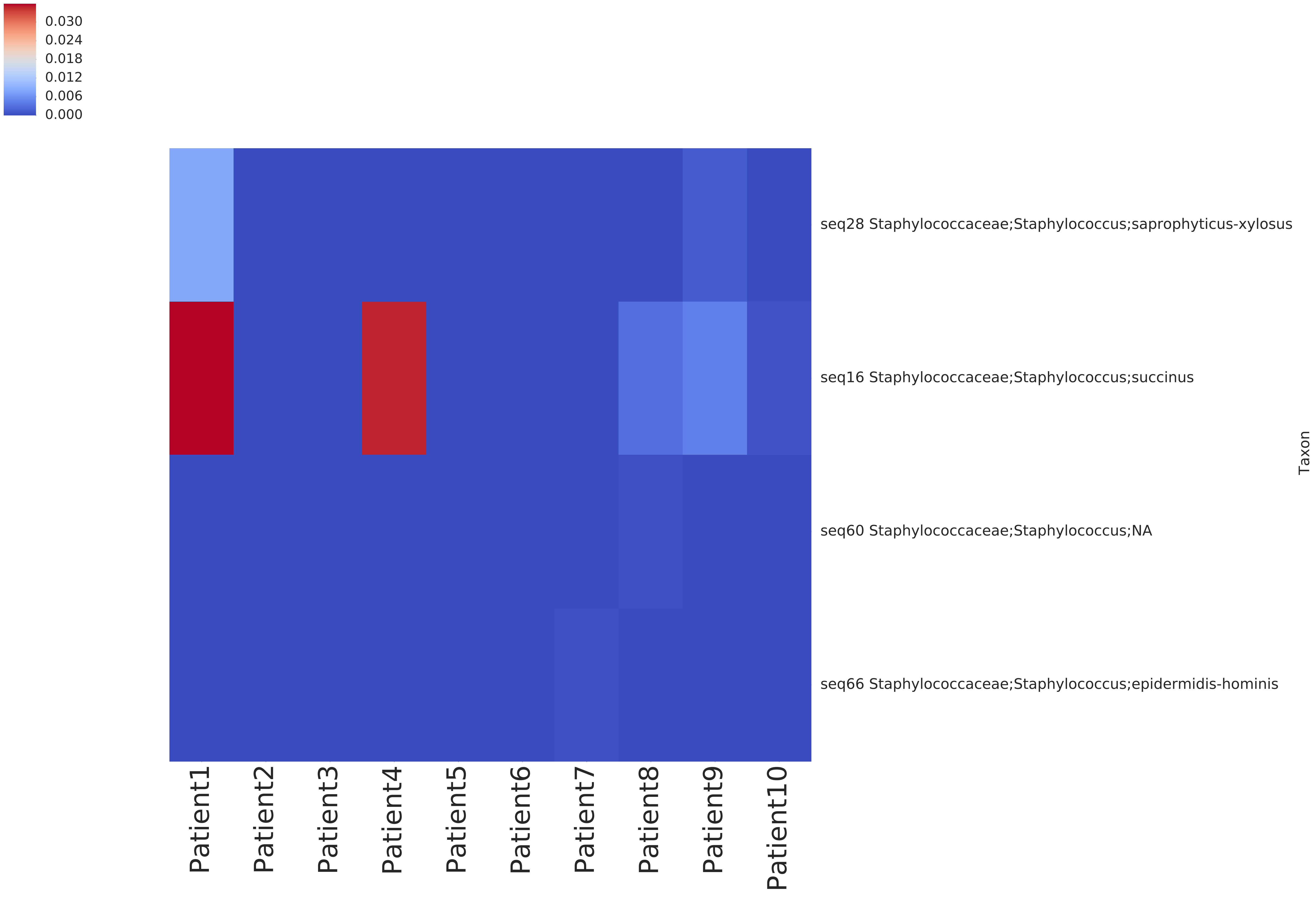
0.030
0.024
0.018
0.012
0.006
0.000
seq28 Staphylococcaceae;Staphylococcus;saprophyticus-xylosus
seq16 Staphylococcaceae;Staphylococcus;succinus
seq60 Staphylococcaceae;Staphylococcus;NA
seq66 Staphylococcaceae;Staphylococcus;epidermidis-hominis
Taxon
Patient1
Patient2
Patient3
Patient4
Patient5
Patient6
Patient7
Patient8
Patient9
Patient10
